# Mosaic Convergence of Rodent Dentitions

**DOI:** 10.1371/journal.pone.0003607

**Published:** 2008-10-31

**Authors:** Vincent Lazzari, Cyril Charles, Paul Tafforeau, Monique Vianey-Liaud, Jean-Pierre Aguilar, Jean-Jacques Jaeger, Jacques Michaux, Laurent Viriot

**Affiliations:** 1 Institut des Sciences de l'Evolution, CNRS UMR 5554, Université de Montpellier 2, Montpellier, France; 2 European Synchrotron Radiation Facility, BP220, Grenoble, France; 3 Institut International de Paléoprimatologie et Paléontologie Humaine, Evolution et Paléoenvironnement, CNRS UMR 6046, Université de Poitiers, Poitiers, France; 4 Ecole Pratique des Hautes Etudes et Institut des Sciences de l'Evolution, CNRS UMR 5554, Université de Montpellier 2, Montpellier, France; 5 Team «Evo-Devo of Vertebrate Dentition», Institut de Génomique Fonctionnelle de Lyon, Université de Lyon, CNRS UMR 5242, INRA, Université Claude Bernard Lyon 1, Ecole Normale Supérieure de Lyon, Lyon, France; University of California Berkeley, United States of America

## Abstract

**Background:**

Understanding mechanisms responsible for changes in tooth morphology in the course of evolution is an area of investigation common to both paleontology and developmental biology. Detailed analyses of molar tooth crown shape have shown frequent homoplasia in mammalian evolution, which requires accurate investigation of the evolutionary pathways provided by the fossil record. The necessity of preservation of an effective occlusion has been hypothesized to functionally constrain crown morphological changes and to also facilitate convergent evolution. The Muroidea superfamily constitutes a relevant model for the study of molar crown diversification because it encompasses one third of the extant mammalian biodiversity.

**Methodology/Principal Findings:**

Combined microwear and 3D-topographic analyses performed on fossil and extant muroid molars allow for a first quantification of the relationships between changes in crown morphology and functionality of occlusion. Based on an abundant fossil record and on a well resolved phylogeny, our results show that the most derived functional condition associates longitudinal chewing and non interlocking of cusps. This condition has been reached at least 7 times within muroids via two main types of evolutionary pathways each respecting functional continuity. In the first type, the flattening of tooth crown which induces the removal of cusp interlocking occurs before the rotation of the chewing movement. In the second type however, flattening is subsequent to rotation of the chewing movement which can be associated with certain changes in cusp morphology.

**Conclusion/Significance:**

The reverse orders of the changes involved in these different pathways reveal a mosaic evolution of mammalian dentition in which direction of chewing and crown shape seem to be partly decoupled. Either can change in respect to strong functional constraints affecting occlusion which thereby limit the number of the possible pathways. Because convergent pathways imply distinct ontogenetic trajectories, new Evo/Devo comparative studies on cusp morphogenesis are necessary.

## Introduction

**Figure 1:**
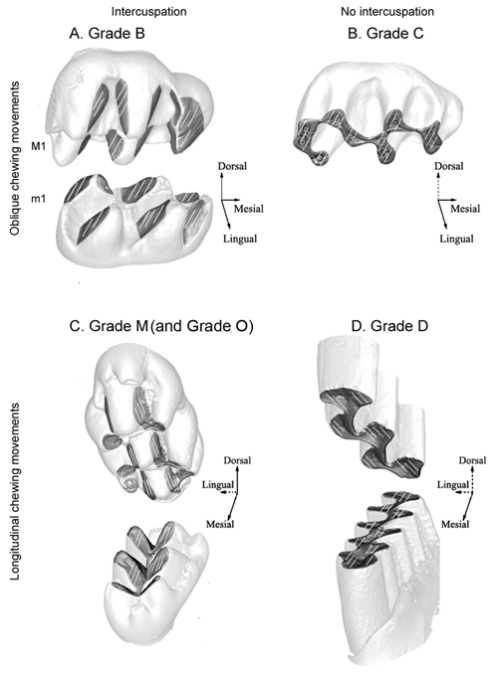
The four morpho-functional grades hypothesized in Muroidea by Butler [9] on first upper and lower molars. First column: grades with cusp interlocking during occlusion. Second column: grades without cusp interlocking during occlusion. First line: grades displaying oblique chewing movements. Second line: grades displaying longitudinal chewing movements. Grey tinted areas on teeth delimitate wear facets while white lines display the orientation of microscratches. Arrows indicate the spatial orientation of the tooth. Full arrows indicate the occurrence of spatial components of the chewing movement in the related direction. Dotted arrows indicate no spatial component of movement in the related direction. Presence of a dorsal component of the chewing movement implies cusp interlocking. Absence of a lingual component of chewing movement implies propalinality. A. Grade B: oblique chewing and cusp interlocking associated with cuspidate tooth crown and cricetine dental plan [9]. B. Grade C: oblique chewing and non cusp interlocking associated with flattened tooth crown and cricetine dental plan [9]. C. Grade M: longitudinal chewing and cusp interlocking associated with cuspidate tooth crown and murine dental plan [9], [11]. Grade O also corresponds to this association. D. Grade D: longitudinal chewing and non cusp interlocking associated with flattened tooth crown and cricetine dental plan [9].

For decades tooth crown morphology in mammals has provided key characters for taxonomy, phylogeny and reconstruction of diet adaptations of past species [1]–[3]. Recent discoveries in the developmental field have hoisted tooth morphology to the rank of privileged model for Evo-Devo studies [4]–[6]. Understanding mechanisms that guided tooth crown morphological changes during evolution therefore constitute a crucial area of investigation common to both paleontology and developmental biology. A first step was achieved when three-dimensional investigations enlightened the adaptive relationships between tooth complexity and diet in mammals [7]. The next step, attained in the present work by combined microwear and topographic analyses, consists of a quantitative study of the relationships between chewing movements and crown morphology as hypothesized in previous analyses [8]–[11]. Due to selection constraints in crown morphological evolution, an effective occlusion has to be maintained in order to ensure functional continuity [9]. Muroid rodents appear as the most relevant model in an investigation of this key point because the superfamily Muroidea (sensu Musser and Carleton [12]) includes about one third of modern mammal biodiversity and their phylogeny is well settled [13]–[15]. Muroid molars also display a huge diversity in terms of cusp arrangement and crown elevation. The cricetine dental plan (e.g. *Cricetus*) illustrates the primitive cusp arrangement of the superfamily [16] with 6 cusps on the first upper molars (M1). The intermediary (e.g. *Dendromus*) and murine (e.g. *Mus*) dental plans show derived conditions which have been reached several times during evolution and display respectively 7 and 8 cusps on M1 [11], [14], [16]. The various functional types of occlusion in muroid molars are characterized by two variables; i) the interlocking of corresponding valleys and summits delimited by cusps rows of opposite teeth [11] and ii) the direction of masticatory movements, which can be oblique or propalinal (longitudinal) within the horizontal plane. Butler [8], [9] used qualitative observations of these variables to define four morpho-functional grades for muroid teeth (Fig. 1). Rodents with grade B (Fig. 1*A*) display oblique chewing movements, cuspidate crowns and cusp interlocking during occlusion [9]. Their cusps bear distinct wear facets and delimit gutters, which allow cusp interlocking. Rodents with grade C (Fig. 1*B*) also masticate with oblique movements. Their molar crowns are nearly flat, without any well-individualized wear facets [9] and they occlude without cusp interlocking. Grade D rodents (Fig. 1*D*) exhibit propalinal movements and flat molar occlusal surfaces with no cusp interlocking [9]. Butler recognized the functional singularity of murine molars, for which the grade M has been recently proposed [11]. This grade (Fig. 1*C*) associates propalinal chewing [8] with cuspidate molars displaying longitudinal gutters which occlude with cusp interlocking [11]. A few members of the muroidea superfamily display cuspidate molars and propalinal movement without longitudinal gutters which limits the longitudinal amplitude of this movement. We defined this new association as grade O (Fig. 1*C*). Grade B is taken to be the primitive condition within Muroidea [10] while Grades C, D and M are taken to be derived conditions [9]. Transitions from one grade to another are supposed to follow unique evolutionary pathways because of the high functional integration of mammalian dentitions. Crown planation was suggested to allow for a rotation of chewing movement documented by the transition from grade B to grade D through grade C [9], [10]. Cusp reshaping inducing a rotation of crown gutters was proposed to explain the origination of grade M from grade B [11]. Nevertheless these hypotheses have never been tested by analyses confronting morphological, functional and phylogenetic data, and validity of masticatory grades has to be confirmed. In what order do the functional and morphological transformations take place? Does functional continuity allow more flexibility than expected by previous studies?

We propose to first of all define new morphological and functional descriptors of tooth crown in order to quantitatively validate proposed muroid molar morpho-functional grades. We will then investigate evolutionary connexions between the different grades by comparing our results with phylogenetic data [13]–[15]. Finally we will discuss the relationship between occlusion and molar morphological changes through evolution. For the purpose of this study we sampled species (See Table 1) representing a significant survey of morphological dental diversity in both extant and extinct muroids. The M1 of 27 species belonging to 11 muroid subfamilies were digitized using X-ray synchrotron microtomography at the European Synchrotron Radiation Facility (Grenoble, France) to compute different topographic maps quantifying various aspects of crown morphology such as elevation and slope. Microwear analyses were also carried out to characterize wear facets and infer chewing movements independently of the morphology.

**Table 1:** Material. For each taxa, subfamily, collection, geographical origin (with locality) and geological age are indicated.

Taxa	Subfamily	Collection	Locality	Relative age
*Acomys dimidiatus*	Deomyinae	COUM	Arabie Saoudite	Extant
*Apodemus dominans*	Murinae	CPUM	Mont-Helene (France)	Pliocene
*Atavocricetodon huberi*	Paracricetodontinae	CPUM	Rigal Jouet (France)	Oligocene
*Brachyuromys ramirohitra*	Nesomyinae	MNHN	Madagascar	Extant
*Cansumys canus*	Cricetinae		China	Extant
*Cricetodon albanensis*	Cricetodontinae	CPUM	La Grive M (France)	Middle Miocene
*Cricetomys sp.*	Cricetomyinae	CPUM	Congo	Pliocene?
*Democricetodon* sp.	Cricetodontinae	CPUM	(France)	Early Miocene
*Dendromus sp.*	Dendromurinae	CPUM	Makapansgat (South Africa)	Middle Miocene
*Dendromus sp.*	Dendromurinae	CPUM	Makapansgat (South Africa)	Pliocene
*Dendromus sp.*	Dendromurinae	CPUM	KA2 (South Africa)	Pliocene
*Deomys ferrugineus*	Deomyinae	MNHN	Gzanjon (Congo)	Extant
*Eliurus webbi*	Nesomyinae	MNHN	Madagascar	Extant
*Eucricetodon hesperius*	Paracricetodontinae	CPUM	Paulhiac (France)	Early Miocene
*Gerbillus dasyurus*	Gerbillinae (Gerbillini)	COUM	Israel	Extant
*Hispanomys sp.*	Cricetodontinae	CPUM	Lo Fournas 6a (France)	Late Miocene
*Hispanomys castelnovi*	Cricetodontinae	CPUM	Castelnou 6 (France)	Middle Miocene
*Ichtyomys hydrobates*	Sigmodontinae	MNHN	Venezuela	Extant
*Macrotarsomys bastardi*	Nesomyinae	MNHN	Madagascar	Extant
*Megacricetodon aunayi*	Cricetodontinae	CPUM	Blanquatère-1 (France)	Early Miocene
*Megacricetodon gregarius*	Cricetodontinae	CPUM	Castelnou 1bis (France)	Late Miocene
*Megacricetodon tautavelensis*	Cricetodontinae	CPUM	Blanquatère-1 (France)	Early Miocene
*Mesocricetus auratus*	Cricetinae	COUM	France	Extant
*Microtus duodecimcostatus*	Arvicolinae	MNHN COUM	France	Extant
*Mus musculus*	Murinae	COUM	France	Extant
*Myocricetodon irhoudi*	Gerbillinae (Myocricetodontini)	CPUM	Pataniak 6 (Maroc)	Middle Miocene
*Myocricetodon ouedi*	Gerbillinae (Myocricetodontini)	CPUM	Oued Zra (Maroc)	Late Miocene
*Myocricetodon parvus intermedius*	Gerbillinae (Myocricetodontini)	CPUM	Pataniak 6 (Maroc)	Middle Miocene
*Mystromys sp.*	Mystromyinae	CPUM	Swartktrans SK (South Africa)	Pliocene
*Neotoma mexicana*	Neotomyinae	MNHN	Mexico	Extant
*Otomys tropicalis*	Otomyinae (Murinae)	MNHN	Omo (Ethiopia)	Extant
*Paracricetodon cadurcense*	Paracricetodontinae	CPUM	Rigal Jouet (France)	Oligocene
*Peromyscus yucatanicus*	Neotomyinae	COUM	Mexico	Extant
*Preacomys sp.*	Deomyinae	ARI	Harasib (Namibia)	Late Miocene
*Progonomys cathalai*	Murinae	CPUM	Montredon (France)	Late Miocene
*Progonomys clauzoni*	Murinae	CPUM	Lo Fournas 16-M (France)	Late Miocene
*Rhagamys*	Murinae	CPUM	Corse	Pliocene
*Rhagapodemus*	Murinae	CPUM	France	Pliocene
*Rotundomys montisrotundi*	Cricetodontinae	CPUM	Lo Fournas 6a (France)	Late Miocene
*Ruscinomys europeus*	Cricetodontinae	CPUM	Layna (Spain)	Pliocene
*Ruscinomys schaubi*	Cricetodontinae	CPUM	Los Mansuetos (Spain)	Late Miocene
*Spalax leucodon*	Spalacinae	MNHN	Irak	Extant
*Sigmodon hispidus*	Sigmodontinae	MNHN	Brésil	Extant
*Stephanomys*	Murinae	CPUM	France	Pliocene
*Typhlomys anereus*	Platacanthomyinae	MNHN		Extant
*Zygodontomys brevicaudata*	Sigmodontinae	MNHN	French Guiana	Extant

COUM: Collection ostéologique de l'Université de Montpellier. CPUM: Collection paléontologique de l'Université de Montpellier. MNHN: Muséum National d'Histoire Naturelle de Paris.

## Results and Discussion

### Establishment of morphological and functional descriptors

**Figure 2:**
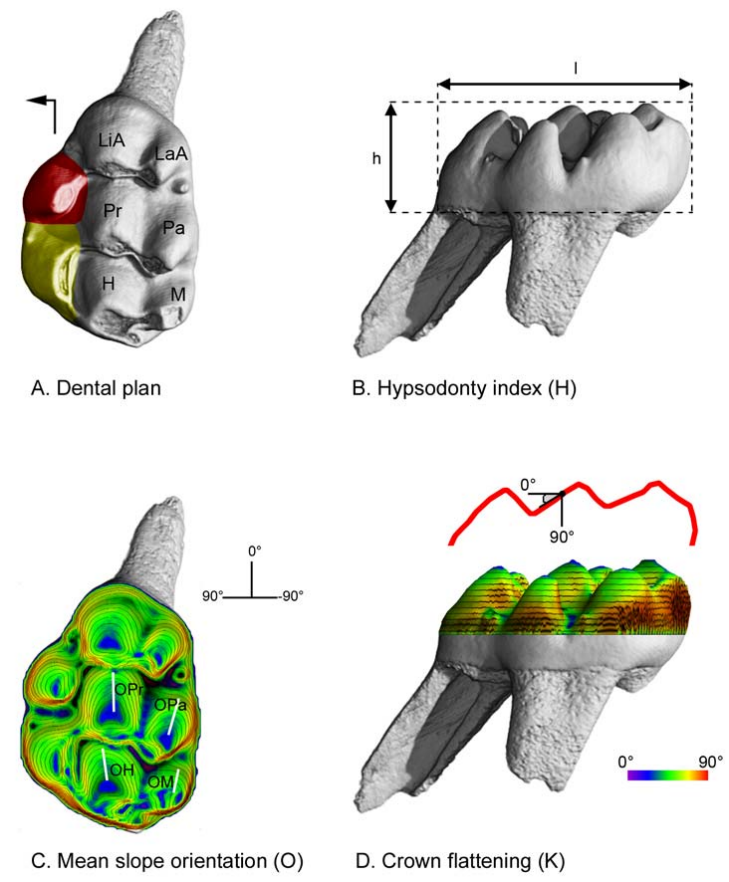
Topographic descriptors of the muroid molar tooth crown shape. A: Dental plans in Muroidea. The cricetine dental plan refers to first upper molar teeth which display the six following cusps: LaA (labial anterocone), LiA (lingual anterocone), Pa (paracone), Pr (protocone), M (metacone) and H (hypocone). The intermediary dental plan refers to the occurrence of one single supplementary lingual cusp (in red). The murine dental plan refers to the occurrence of two or more supplementary lingual cusps (in red and in yellow). The black arrow indicates the mesial and lingual sides of the tooth. B: Hypsodonty Index (H). l: length of the tooth crown. h: high of the tooth crown. H = h/l. C: Average orientation (O) of the lowest slope of the four main cusps of the muroid first upper molar. The orientations of the lowest slopes are observed thanks to a slope colour map with superimposed topographic contour lines (computed with Surfer for Windows). OPa: lowest slope orientation of the paracone. OPr: lowest slope orientation of the protocone. OM: lowest slope orientation of the metacone. OH: lowest slope orientation of the hypocone. O = (OPa+OPr+OM+OH)/4. D: Crown flattening index (K). K refers to the global shape of the crown topography (red line) and is calculated as the kurtosis of the distribution of the crown slope values provided for each node (black point) of the computed slope maps (3D slope colour map with superimposed topographic contour lines, computed with Surfer for Windows).

**Figure 3:**
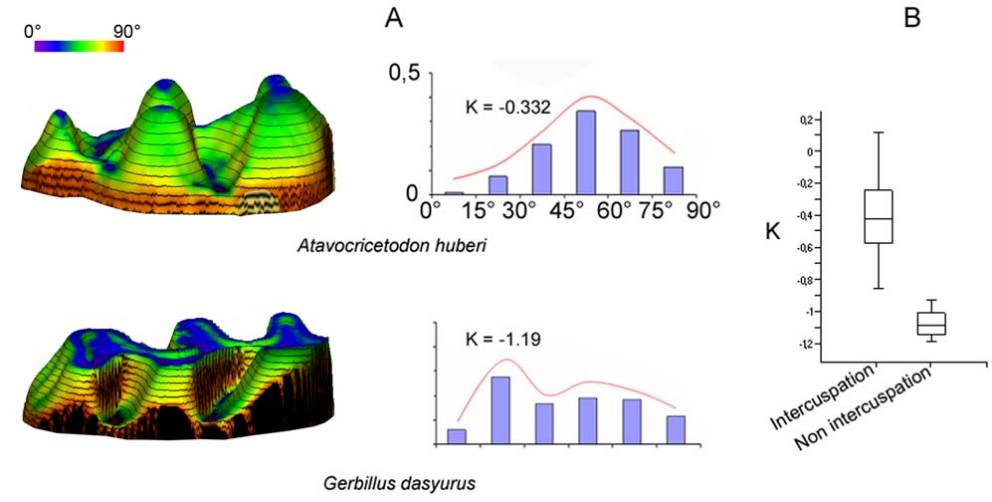
Topographic investigation of tooth crown planation in mammals. A: Topography of the left first upper molars of *Atavocricetodon huberi* and *Gerbillus dasyurus*, and the associated histograms of slope value distribution on the tooth crown, with values of Kurtosis K calculated for both taxa (3D slope map with superimposed contour lines computed with Surfer for Windows). B: Box plots showing K value distribution of Muroidea displaying cusp interlocking (n = 23, mean = −0,415) and Muroidea displaying no cusp interlocking (n = 4, mean = −1,08). Mean K values are significantly different between both groups (Student t test: P<0,001).

Occlusion in Muroidea is functionally characterized by the occurrence of cusp interlocking and the direction of chewing movements (CD). Discontinuous wear facets observable on lightly worn teeth testify to the occurrence of cusp interlocking or intercuspation (Fig. 1*A and 1B*). Continuous wear facets are produced along a unique occlusal plane and correspond to non cusp interlocking. The CD value is the angle between the longitudinal tooth row axis and the orientation of microwear scratches [10]. The morphology of molar crown in mammals can be characterized by numerous topographic parameters [e.g. 7], [11], [17], [18]. The present work is focused on the dental plan, the average cusp lowest slope orientation O, the degree of crown levelling K and the hypsodonty index H (Fig. 2). Molars of studied species were assigned to cricetine, murine or intermediary dental plans depending on the number and the arrangement of cusps and crests found (Fig. 2*A*). The value of O is hypothesized as being related to chewing direction. This descriptor refers to cusp individual shape and more precisely to the lowest slope average orientation in the four main cusps of muroid molars (protocone, hypocone, paracone and metacone, Fig. 2*C*). The orientation of the lowest slope of the protocone and hypocone has already been shown to be correlated to the direction of chewing in cuspidate muroid molars [11]. The K parameter refers to the crown surface global shape (Fig. 2*D*) and is estimated by the kurtosis of the distribution of crown slope values provided by computed slope maps (Fig. 3*A*). It measures the “peakedness” of this distribution. Distributions with K values around 0 are unimodal and indicate a cuspidate crown with rounded cusps (Fig. 3*A*). Distributions with K inferior to −1 are bimodal with an abundance of extreme slope values and indicate angular cusps and a crown with a nearly flat occlusal surface delimited by steep slopes (Fig. 3*A*). The hypsodonty index H refers to the relative crown elevation (crown height/crown length, Fig. 2*B*) [19]. The occurrence of cusp interlocking, the dental plan and the values of CD, K, O and H for each taxon are presented in Table 2.

**Table 2:** Values of functional and topographic crown descriptors for the left first upper molar of each taxa.

Taxa	cusp interlocking	CD (N)	Dental plans	K	O	H
*Acomys dimidiatus*	YES	0,0 (2)	murine	−0,196	4,75	0,423
*Apodemus dominans*	YES	−0,2 (3)	murine	−0,767	7,25	0,423
*Atavocricetodon huberi*	YES	68,3 (2)	cricetine	−0,332	69	0,436
*Cricetodon albanensis*	YES	69,4 (11)	cricetine	−0,578	45,8	0,427
*Cricetomys* sp.	NO	1,2 (2)	murine	−1,09	19,8	0,492
*Democricetodon* sp.	YES	61,2 (4)	cricetine	−0,425	60,3	0,435
*Dendromus* sp.1	YES	2,1 (3)	intermediary	−0,265	15,3	0,351
*Dendromus* sp.3	YES	3,8 (3)	intermediary	−0,491	6,75	0,376
*Deomys ferrugineus*	YES	1,1 (3)	intermediary	−0,471	2,5	0,430
*Eucricetodon hesperius*	YES	64,2 (2)	cricetine	0,065	64	0,447
*Gerbillus dasyurus*	NO	0,0 (1)	cricetine	−1,19	14	0,418
*Hispanomys castelnovi*	YES	70,7 (5)	cricetine	−0,777	47,3	0,415
*Megacricetodon aunayi*	YES	49,0 (1)	cricetine	−0,244	62	0,373
*Megacricetodon tautavel.*	YES	38,0 (1)	cricetine	−0,485	49,3	0,460
*Mesocricetus auratus*	YES	69,1 (1)	cricetine	−0,86	72,8	0,411
*Microtus duodecimcostatus*	NO	−1,0 (2)	cricetine	−1,1	14	1,261
*Mus muscuslus*	YES	0,0 (1)	murine	−0,397	11,3	0,360
*Myocricetodon irhoudi*	YES	27,0 (1)	cricetine	−0,256	31	0,381
*Myocricetodon ouedi*	YES	2,5 (3)	cricetine	−0,637	30,3	0,387
*Myocricetodon parvus int.*	YES	45,6 (3)	intermediary	−0,247	37,5	0,351
*Mystromys* sp.	YES	71,5 (2)	cricetine	−0,507	47	0,480
*Peromyscus yucatanicus*	YES	50,0 (1)	cricetine	−0,678	46,5	0,444
*Preacomys* sp.	YES	1,1 (1)	murine	−0,153	7	0,377
*Progonomys cathalai*	YES	−3,0 (3)	murine	−0,386	1,25	0,403
*Progonomys clauzoni*	YES	−1,0 (1)	murine	0,112	−3	0,450
*Rotundomys montisrotundi*	NO	33,0 (4)	cricetine	−0,93	42	0,430
*Ruscinomys schaubi*	YES	59,7 (25)	cricetine	−0,572	42,8	0,605

CD: chewing direction value in degrees (with N the number of measured individuals); K: kurtosis of the crow slope distribution. O: average cusp lowest slope orientation value in degrees. H: hypsodonty index. Cusp interlocking is inferred from the observation of wear facets. Cusp interlocking is associated with discontinuous wear facets.

### Quantitative morpho-functional analysis

Several correlations between the functional and morphological descriptors are observed. Muroid molars with continuous wear facets and flattened crowns have K values significantly lower than those of the molars with discontinuous wear facets (Fig. 3*B*). Non occurrence of cusp interlocking is thus linked to flattened crowns. Such crowns display continuous occlusal planes. The correlation matrix between CD, K, O, H and the results of various tests are given in Table 3. We can see that CD is strongly correlated with O (r = 0.91; P<0.001), but neither with H nor K (Table 3). While O and CD are known to be correlated when cusp interlocking occurs [11], the present results indicate that O is also correlated to CD in case of non cusp interlocking. Chewing direction is thus nearly always parallel to the average orientation of the cusp lowest slopes while it is independent from crown elevation and crown flattening.

**Table 3:** Correlation matrix between the morpho-functional descriptors CD, K, O and H.

	H	O	K
CD	(−0.093; 0.644; 0.513)	**(0.912 ; 3.47^E^−11; 8.57^E^−6)**	(0.06; 0.764; 0.682)
K	(−0.398; 0.04; 0.117)	(−0.02; 0.919; 0.521)	
O	(−0.077; 0.702; 0.323)		

Each cell of the table displays function (r, P1, P2). r is the coefficient of correlation of Pearson and P1 is the value associated with the probability test of uncorrelation between variables. P2 is the value of probability of uncorrelation between variables associated with non parametric Spearman's Rank Correlation Test. The number of individuals n = 27. Bold values: statistically correlated values. Regular values: not statistically correlated values. CD: chewing direction; K: kurtosis of the crow slope distribution. O: average cusp lowest slope orientation of protocone, paracone, hypocone, and metacone. H: hypsodonty index.

**Figure 4:**
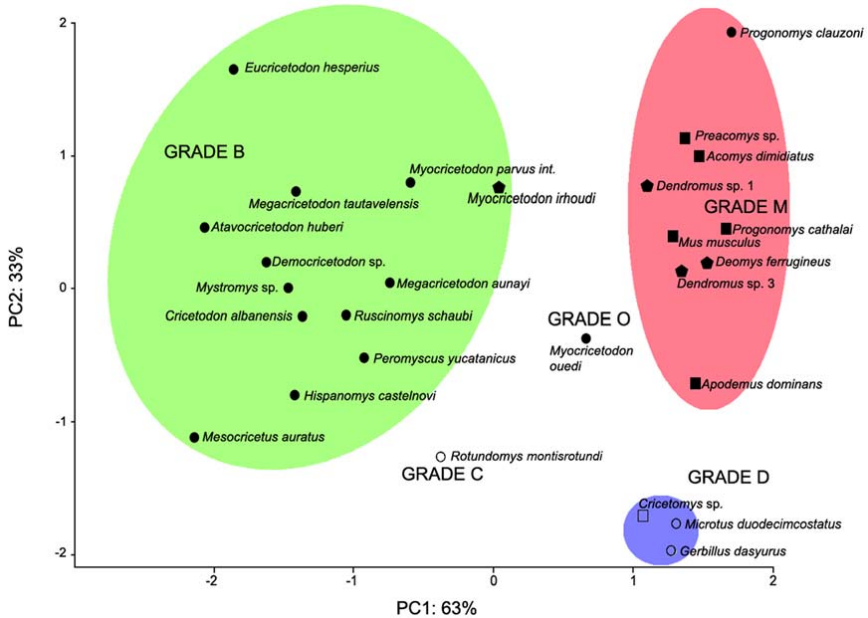
Discrimination of the masticatory grades in Muroidea (27 taxa). PCA performed on morpho-functional parameters CD, K, O and H. PC1 (63% of variance) is strongly supported by the direction of chewing (CD) and by the average orientation of main cusps lowest slopes (O). PC2 (33% of variance) is strongly supported by crown flattening (K). The green area indicates grade B, red area grade M and blue area grade D. Grades C and O are situated in intermediary positions. Cricetine dental plans are shown by circles, intermediary plans are indicated by pentagons and murine plans are represented by squares. Full points indicate cusp interlocking (discontinuous wear facets), while empty points indicate no cusp interlocking (continuous wear facets).

A principal component analysis (Fig. 4) was performed on the linear correlation matrix of CD, K, O, and H (Table 3). PC1 is strongly supported by CD and O, while PC2 is mainly related to K. The PCA shows that three main morpho-functional groups are distinguished among Muroidea (Fig. 4): i) a group corresponding to grade B with cuspidate crowns, oblique orientation of lowest cusp slope, and oblique chewing movements,; ii) a group corresponding to grade D with flattened crowns, longitudinal orientation of lowest cusp slope, and propalinal chewing movement; iii) a group corresponding to grade M with cuspidate crowns, longitudinal orientation of cusp lowest slopes, and propalinal chewing movements. Two taxa show intermediary situations. *Rotundomys* displays a flattened crown and an oblique chewing direction which corresponds to a grade C. *Myocricetodon ouedi* has a cuspidate crown, shows an oblique orientation of lowest cusp slope, and exhibits an occlusion characterized by cusp interlocking with propalinal movements which corresponds to a grade O.

The PCA validates the morpho-functional grades proposed for Muroidea [8]–[11] but refines their description with regards to those previously proposed (see in Fig. 1): Grade B, C and O are associated with the cricetine dental plan; M to the intermediary and murine plans; and D to the cricetine and murine plans (Fig. 4). Masticatory grades and dental plans are thus not strictly correlated in Muroidea. With lowest cusp slopes displaying similar orientations to those of the grade B, Grade C can be now morphologically distinguished from Grade D. However, no crown topographic parameter distinguishes grades B and O, which both display cusp interlocking, but have different directions of masticatory movements. Grade O Muroidea present propalinal chewing movements but in this case opposite cusps can not slide in longitudinal gutters. Therefore the longitudinal amplitude of the chewing movements in these rodents is limited, wear facets being sub-vertical. The various morpho-functional grades in muroid rodents can all be characterized by microwear patterns (wear facet continuity and microscratches orientation) except for the rare Grade O. We therefore assigned a putative morpho-functional grade here for 35 supplementary muroid rodent taxa according to their microwear pattern (Table 4).

**Table 4:** Microwear patterns of various muroid rodents.

Taxon	Subfamily	Cusp interlocking	CD (N)	Source	Inferred masticatory grade
*Brachyuromys ramirohitra*	Nesomyinae	NO	41° (1)	P.W.	C
*Cansumys canus*	Cricetinae	YES	65° (2)	P.W.	B
*Eliurus webbi*	Nesomyinae	NO	4°(1)	P.W.	D
*Ichtyomys hydrobates*	Sigmodontinae	NO	77°(1)	P.W.	B
*Macrotarsomys bastardi*	Nesomyinae	NO	36°(1)	P.W.	B
*Megacricetodon gregarius*	Cricetodontinae	NO	44°(3)	P.W.	B
*Neotoma mexicana*	Neotomyinae	YES	5° (1)	P.W.	D
*Otomys tropicalis*	Otomyinae (Murinae)	NO	3° (1)	P.W.	M
*Paracricetodon cadurcense*	Paracricetodontinae	NO	80° (4)	P.W.	B
*Rhagamys orthodon*	Murinae	YES	−2° (2)	P.W.	D
*Rhagapodemus* sp.	Murinae	NO	0° (1)	P.W.	M
*Ruscinomys europeus*	Cricetodontinae	NO	55° (3)	P.W.	B
*Spalax leucodon*	Spalacinae	YES	42° (1)	P.W.	C
*Sigmodon hispidus*	Sigmodontinae	YES	28° (2)	P.W.	C
*Stephanomys sp.*	Murinae	NO	−1° (4)	P.W.	M
*Typhlomys anereus*	Platacanthomyinae	YES	26° (1)	P.W.	C
*Zygodontomys brevicaudata*	Sigmodontinae	NO	54° (1)	P.W.	B
*Apodemus sylvaticus*	Murinae	NO	0°	Charles et al. (10)	M
*Arvicanthis ansorgei*	Murinae	NO	0°	Charles et al. (10)	M
*Arvicola terrestris*	Arvicolinae	YES	0°	Charles et al. (10)	D
*Calomyscus bailwardi*	Calomyscinae	NO	« anterolingual »	Wahlert (20)	B
*Cricetops dormitor*	Cricetopinae	NO	«anterolingual»	Wahlert (20)	B
*Cricetus cricetus*	Cricetinae	NO	60°	Charles et al. (10)	B
*Eumys elegans*	Eumyinae	NO	55°	Butler (21)	B
*Gerbillus minutus*	Gerbillinae	YES	0°	Tong (21)	D
*Lophiomys imhausii*	Lophiomyinae	NO	«anterolingual»	Wahlert (20)	B
*Holochilus* sp.	Sigmodontinae	YES	15°	Butler (8)	C
*Meriones crassus*	Gerbillinae	YES	0°	Charles et al. (10)	D
*Mesocricetus auratus*	Cricetinae	NO	60°	Charles et al. (10)	B
*Micromys minutus*	Murinae	NO	0°	Charles et al. (10)	M
*Myocricetodon* cf. *irhoudi*	Myocricetodontini	NO	22°	Tong (21)	B
*Myospalax fontanieri*	Myospalacinae	YES	2°	Charles et al. (10)	D
*Potwarmus thailandicus*	Dendromurinae?	NO	0°	Tong (21)	M
*Protatera* sp.	Gerbillinae	YES	0°	Tong (21)	D
*Saccostomus campestris*	Cricetomyinae	YES	5°	Charles et al. (10)	D

The subfamily is given for each taxa as well as the occurrence of cusp interlocking, the direction of mastication in degrees (with N the number of measured individuals when realized in the present work) and the occurrence of cusp interlocking measured in the present work (P.W.) or already appearing in other publications. We propose a masticatory grade for each taxa based upon the observation of microwear pattern.

### Morpho-functional grades and phylogeny

**Figure 5:**
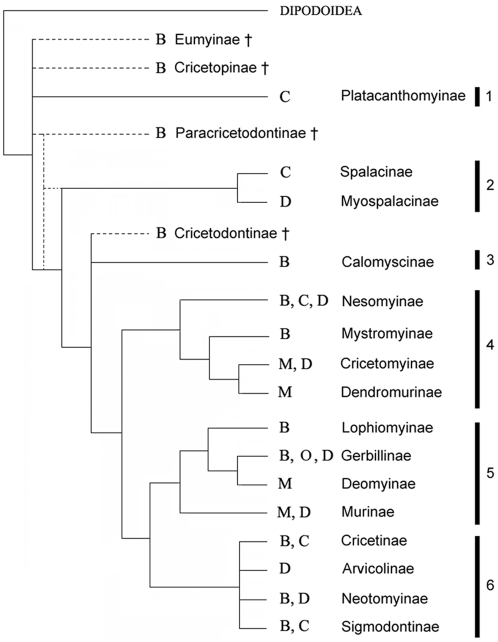
Morpho-functional chewing grades and muroid phylogeny. This phylogeny is adapted from complementary results from the three most recent molecular phylogenies [13]–[15] and some palaeontological hypotheses [22]–[25]. The phylogenetic position of Platacanthomyidae has not been investigated yet with molecular data. We inferred the chewing grade of taxa whose direction of chewing has already been published or was measured in this study (See Table 4). B: grade B; C: grade C; D: grade D; O: grade O; M: grade M; †: fossil taxa. 1: Platacanthomyidae; 2: Spalacidae; 3: Calomyscidae; 4: Nesomyidae. 5: Muridae; 6: Cricetidae.

Comparison of our results with molecular and palaeontological data relative to muroid phylogeny [13]–[15], [22]–[25] (Fig. 5) confirms that grade B is a primitive condition within Muroidea. It also reveals that the derived grades (C, M and D) were independently reached in several cases. Such results were to be expected because of the numerous cases of dental homoplasy observed in the course of muroid evolution revealed by a previous study combining dental characteristics and molecular data [14]. The emergence of grade D appears to be the most frequently achieved within the muroid radiation (at least 7 times) while grade C appeared at least 5 times and grade M at least 3 times (Fig. 5). Grade O up to now has only been recognized in one subfamily.

### Dental morpho-functional evolution in Muroidea

**Figure 6:**
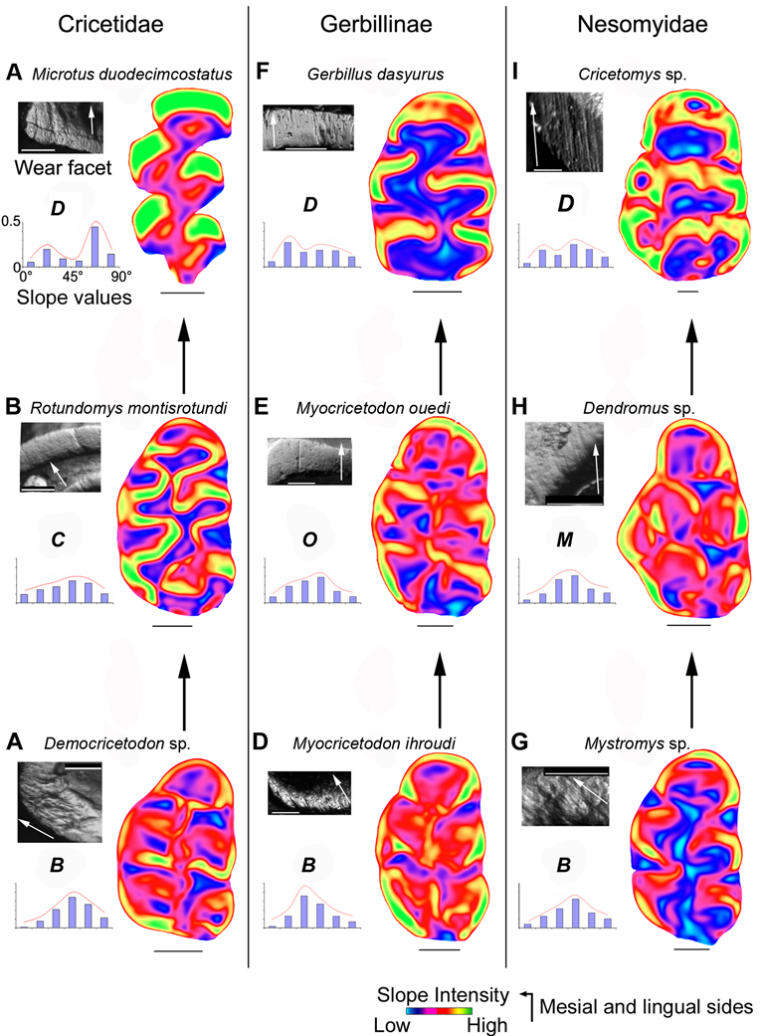
Convergent morpho-functional evolution revealed by microwear pattern and topographic slopes crown maps in three muroid lineages. Cricetidae (A. *Democricetodon* sp., B. *Rotundomys motisrotundi*, C. *Microtus duodecimcostatus*), Gerbillinae (D. *Myocricetodon irhoudi*, E. *Myocricetodon ouedi* and F. *Gerbillus dasyurus*) and Nesomyidae (G. *Mystromys* sp., H. *Dendromus* sp. 2 and I. *Cricetomys* sp.). The morpho-functional grade (B, C, O, M or D in bold) is inferred from crossed quantitative interpretations of crown topography and microwear pattern on left M1. In the upper left quarter is a picture of a wear facet for each species. The white arrow indicates the mean direction of microscratches corresponding to the direction of chewing. White scale bar: 100 µm. A colour slope map displaying the orientation of the cusps lowest slopes is presented on the right half of the diagram for each species. Black scale bar: 500 µm. The histogram of distribution of crown slopes is presented in the lower left quarter. Unimodal histograms (Kurtosis superior to −1) indicate cuspidate crowns, with predominant intermediary slope values associated with round cusps. Bimodal histograms (Kurtosis inferior to −1) indicate flattened crowns, with predominant extreme slope values associated with angular cusps.

The morpho-functional modifications which accompanied grade-to-grade transitions can be reconstructed using fossil record and replaced in the robust muroid phylogenetic context. Several examples of convergent evolutions leading from grade B to grade D through distinct intermediary grades are illustrated here by the Cricetidae (e.g. Arvicolinae), Gerbillinae (e.g. Gerbillinae), and Nesomyidae (e.g. Cricetomyinae). Among Cricetidae, the subfamily Arvicolinae (e.g. *Microtus* Grade D, Fig. 6*C*) emerged during the Late Miocene radiation of “microtoid cricetids” [22] like *Rotundomys* (Grade C, Fig. 6*B*), which originated from Cricetodontinae like *Democricetodon* [23] (Grade B, Fig. 6*A*). In this case the transition from grade B to grade D is accomplished by a transition through grade C. Such a transition is also observed in Nesomyinae and Spalacidae (Fig. 5). Extant Gerbillinae like *Gerbillus* (Grade D, Fig. 6*F*) emerged from Miocene taxa like *Myocricetodon irhoudi* (Grade B, Fig. 6*D*) through intermediary forms like *M. ouedi* [24] (Grade O, Fig. 6*E*). In this lineage, grade D is reached by a transition through grade O. Among Nesomyidae, the Cricetomyinae (e.g. *Cricetomys* Grade D, Fig. 6*I*) display a murine dental plan and constitute the sister group of the Dendromurinae [13]–[15] (e.g. *Dendromus*, Grade M, Fig. 6*H*) which are showing an intermediary plan. Cricetomyinae and Dendromurinae share a common ancestor with the genus *Mystromys* [14] (Grade B, Fig. 6*G*) which displays a cricetine plan. These phylogenetical relationships suggest that grade D has been reached here by a transition through the grade M and accompanied the transition from cricetine to murine dental plan. A similar transition is also observed in Murinae (Fig. 5). However, the emergence of grade D in rodents appears to be more frequently reached via a transition involving grade C. Indeed, not only does it occur at least 3 times in Muroidea (Fig. 5), but it also appears in other groups of rodents as Dipodoidea [10].

**Figure 7:**
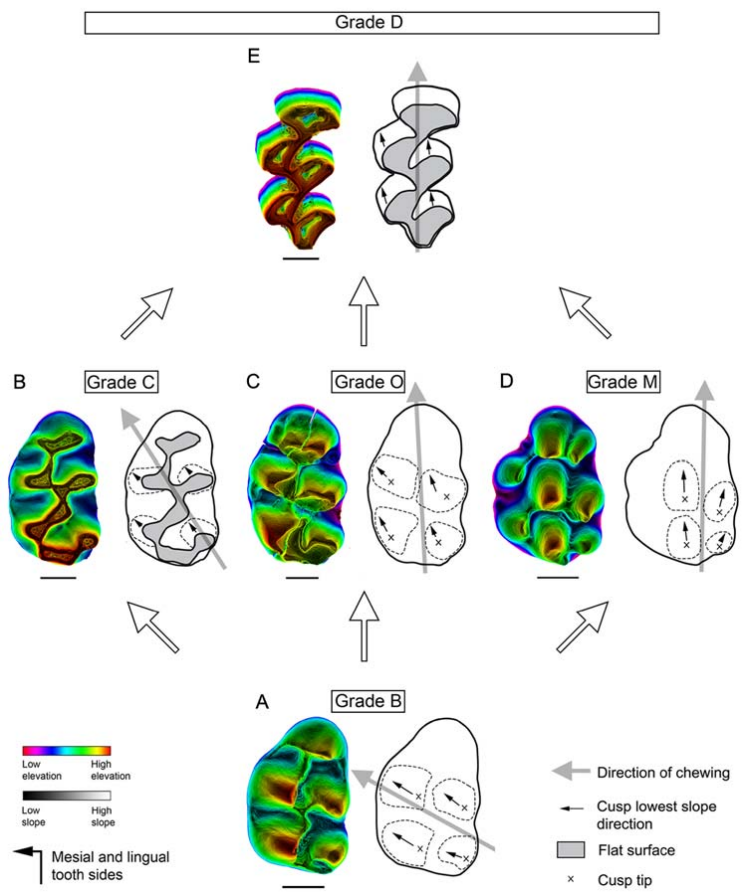
The five morpho-functional grades recognized in the present work in muroid left M1 along with their potential evolutionary relationships. For each grade, a computed 3D rendering with superimposed colour topographic map is presented on the left. The colour code refers to the elevation while the grey-level code refers to the slope value. Scale bar: 500 µm. The topologic map presented on the right indicates the main cusp tip (crosses) or their flattened surface (grey areas), the outline of the four main cusps (dotted lines), the orientation of their lowest slope (black arrow) and the direction of chewing (grey arrow). A. Grade B: oblique masticatory movements and cusp interlocking associated with cuspidate crown, oblique cusp lowest slope and cricetine or intermediary dental plan. B. Grade C: oblique masticatory movements and non cusp interlocking associated with flattened crown, oblique cusp lowest slope and cricetine dental plan. C. Grade O: longitudinal masticatory movements and cusp interlocking associated with cuspidate crown, oblique cusp lowest slope, and cricetine plan. D. Grade M: longitudinal masticatory movements and cusp interlocking associated with cuspidate crown, longitudinal cusp lowest slope, and murine or intermediary dental plans. E. Grade D: longitudinal masticatory movements and non-cusp interlocking associated with flattened crown, longitudinal cusp lowest slope, and murine or cricetine dental plan. During muroid radiation, Grades C, O, and M emerged from grade B and independently gave rise to grade D. The example chosen to illustrate the grades are not phylogenetically related.

All these examples emphasize that each transition between the various morpho-functional grades can be explained by moderate morpho-functional modifications. Transitions from grades B to C or from grades M to D require a crown planation (Fig. 7). This modification results from a progressive change in the shape of cusps and crests whose sides tend to progressively become more and more vertical. Crown planation results in the loss of cusp interlocking although the direction of chewing is preserved. Transition from grades C to D only involves a change of cusp lowest slope orientation (Fig. 7) and thus appears morpho-functionally simple because a flat occlusal surface allows both oblique and propalinal masticatory movements. Contrary to this however, transition from grades B to M implicates the preservation of a cuspidate crown [9], [11] (Fig. 7). Rotation of the chewing movements occurs simultaneously with changes in the direction of the cusp lowest slope: new-shaped cusps delimit new gutters where cusps of the opposite tooth can slide longitudinally during occlusion [11]. Lastly, neither crown planation nor significant changes in cusp morphology occur during the transition from grades B to O (Fig. 7). This example emphasizes that a rotation of chewing movements can occur prior to any cusp morphological change, and could possibly drive them during the course of evolution.

Two quite different ways to get to grade D are emphasized. The pathways involving grades O and M alter first chewing direction (and cusp slopes for Grade M) and only afterwards crown planation takes place. In contrast, the transition involving grade C changes features in a reverse order. Thus there is a mosaic evolution of convergent tooth morphology in Muroidea. From this, we can state that direction of chewing and crown shape are partly decoupled in evolution and that either can change respecting the functional continuity.

Our results indicate that the radiation of muroid rodents is characterized by multiple parallel and convergent evolutions of the molar crown, originating from an ancestor characterized by grade B molars. The small number of morpho-functional grades and the functional continuity displayed by each convergent evolutionary pathway emphasize the strong functional constraints required by the preservation of an efficient occlusion. These constraints can partially explain the similarity of dental patterns observed among extant species [14]. Propalinal chewing and flat crown conditions could have also been promoted in relation with some functional advantages. Food processing in primitive Muroidea, accompanied by transverse mandibular movement (Grade B), occurs alternatively on either side [20]. By comparison, simultaneous mastication of both jaws is only observed in some Muroidea. These Muroidea display parallel left and right rows of cheek teeth, longitudinal chewing motion and relatively flat occlusal surface [26]. Such differences can be linked to a better mastication efficiency. Illustrating such a situation, Muroidea displaying Grade D such as Arvicolinae and some Murinae are known to simultaneously masticate food bilaterally using an anterior jaw shift [27], [28]. Emergence of grade D in Muroidea could thus have promoted simultaneous mastication of both jaws. Compared to the primitive condition, Grade D condition also displays a simplified chewing motion with one single phase since all the wear facets are horizontal and connected. Such a number of iterative evolutions can also be explained by a presumed relative simplicity of the required changes in the tooth developmental program. The shape of cusps, apart from enamel thickness, is largely due to the relative growth of the inner enamel epithelium and the underlying mesenchyme [29]. However the involved molecular mechanisms are not clearly identified as yet. Epithelial clusters of non-dividing cells known as enamel knots express several signalling molecules, and thus participate in regulating the formation of both crown base and occlusal elements. The spatial patterns of enamel knots predict the species-specific cusp spatial arrangements, cusp relative sizes and cusp numbers [4], [29]. Up to the present cusp shape anomalies have been demonstrated to be related only rarely to specific mutations [30] and further studies will have to determine the exact role of enamel knot signalling in patterning of crown element and most notably cusp shaping. The convergent evolutions discussed in the present work imply however distinct ontogenetic trajectories and different Evo/Devo studies to decipher each of them.

Several aspects of tooth topography (O, K) are strongly related to the functional parameters of occlusion (CD, cusp interlocking). Other aspects have been proved to be related to diet by complexity analysis such as the number of breakage sites on a tooth [7]. Combining our cusp morphology descriptors with crown complexity analysis could subsequently allow a very precise integrated study of muroid tooth morphology and function. Such a complete approach would constitute a new toolset to understand the evolutionary relationships between dietary habits and functional features as intercuspation and direction of chewing in rodents. Because it is an homology-free method; it could also be applied for similar investigations in other mammalian groups displaying high diversification of molar morphology, such as carnivorans [7], marsupials, ungulates and primates.

## Materials and Methods

### Microwear pattern analysis

Dental abrasion on fossil teeth is the result of mastication of items of food that were consumed during the last days prior to the death of the animal [31]. Among the different food items, grasses and related plants leave numerous scratches on enamel dental facets [32], [33] because of the high concentration of silica phytoliths in their cell walls [34]. Such scratches cannot be generated by attrition, because they only result from the friction of objects clamped between both equivalent facets. In rodents the orientation of these microwear scratches, whatever their size, indicates the direction of jaw movement during chewing [8]–[10]. Numerical values indicating the direction of the chewing movements (CD) in rodents can be thus obtained by measuring angles between scratch directions and the axis of the jugal tooth row [10].

Scratch orientation does not significantly vary among the distinct facets of a muroid molar and among the various molars of the same row in Muroidea [10]. For this study we took into account not only tooth rows of extant rodent complete skulls but also fossil isolated teeth. In fact complete skulls of fossil taxa are rare, while wear facets are frequently damaged during tooth transport occurring before the fossilization and during fossilization processes. We therefore used all the available molar teeth and wear facets of a species to obtain significant data. Dental elements were first of all cleaned with alcohol and acetone in order to remove dirt and glue from the occlusal surface. Then casts of the teeth were made using polyvinylsiloxane and transparent epoxy resin which was heated at 30°C during 12 hours. Pictures of enamel facets were then digitized in 256 grey levels using ×60, ×80 or ×100 objective (depending on specimen size) with transmitted-light stereomicroscope and CCD camera. The final step was to measure the angle between the scratch and the axis of the jugal tooth row for all the scratches found on a tooth. In case of isolated teeth, we used the mean of the directions of the labial side and lingual side of each tooth to approximate the axis of the jugal tooth row. A mean striation angle value was then calculated for each tooth. The CD value of one species was calculated as the mean of the values obtained for each measured tooth of this species.

Specific direction of mastication CD in Muroidea varies from −5° to 80°. A CD value of 0° indicates a strictly propalinal movement and a value of 90° would indicate a strictly transversal movement. For positive values, the direction deviates toward the lingual side from the mesial direction.

### Topographic tooth crown analysis

We calculated three tooth crown topographic descriptors: the degree of crown planation (K), the mean orientation of the lowest slope of the cusps (O) and the hypsodonty index (H). We performed experiments on rodent molars using X-ray synchrotron microtomography at the European Synchrotron Radiation Facility (ESRF, Grenoble, France). These experiments were carried out on the beamlines ID19 and BM05 with voxel sizes of 2.8, 5.06 and 7.46 µm using moderate propagation phase contrast and a monochromatic X-ray beam at energy levels from 25 to 30 keV. Ring artefacts on reconstructed tomographic slices at 2.8 µm were removed by using a specific automatic script developed by P.T. for the Photoshop 7.0 software (Adobe system, Inc., San Jose, California, USA) [35], [36]. Virtually 3D reconstructed teeth were orientated with VGStudiomax (Volume Graphics, Heildelberg, Germany) according to the cervix plan method [11].

Grey level topographic maps have been computed from these normalized stacks with Photoshop script presented in [11]. These maps provide 8-bit grey level elevation encoding of tooth occlusal morphology. Standardized in size and then converted in. txt format with Image J (http://rsb.info.nih.gov/ij/), they can be used as XYZ data files in Surfer for Windows (Golden Software, Inc) and interpolated as regular grids of points. Terrain slope calculus was performed on those grids. Slope was reported in degrees from 0° (horizontal) to 90° (vertical) at any grid node of the tooth surface. For a particular point on the surface, the terrain slope in Surfer is based on the direction of steepest descent or ascent at that point. Univariate statistics of the crown slope distributions were then computed. The Kurtosis (K) of slope distribution for each tooth was then calculated as below.
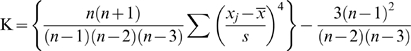
s is the standard deviation of the sample.

For an optimal rendering, we also computed colour slope maps and 3D rendering with superimposed colour topographic maps from normalized stacks with the Photoshop script proposed in [11]. Colour slope maps allow the measurement of the orientation of the lowest cusp slopes of the four main cusps: protocone (OPr), hypocone (OH), paracone (OPa) and metacone (OM). OPr and OH have been proved to be indicators of the orientation of the plane of symmetry of protocone and hypocone in cuspidate crowns, revealing the orientation of the gutters allowing cusp interlocking [11]. Values measured with Photoshop are given in degrees. A value of 0° indicates a longitudinal direction and a value of 90° indicates a transversal direction. For positive values, the direction deviates toward the lingual side from the mesial direction (Fig. 2*C*). Each measurement has been made ten times. The measure error is ±2°. O is the mean of OPr, OH, OPa, and OM. 3D rendering with superimposed colour topographic maps provide a grey-level encoding of slope values combined with a colour encoding of elevation values.

Even though general slope measurements are relatively robust to tooth wear in primates [37], [38], crown topography is known to change dramatically over the course of the cuspidate rodents' life [39], [40]. To obtain functionally comparable measurements of tooth shapes in our analysis, we therefore selected for each species one specimen exhibiting only light to moderate wear, in accordance with the age/wear classes established with discrete dental morphological criteria in Murinae (Fig. S1, Text S1) [39], [40]. Studying the influence of wear on K and O in a single cuspidate species, we indeed highlightened that these descriptors do not significantly vary between age/wear classes I to III (Fig. S1, S2, Tables S1, S2, Text S1) which correspond to light to moderate wear. On the other hand from wear class IV, the wear is so important that a cuspidate species displays K values non significantly different from a species with flat crown (Fig. S2, Table S1, Text S1). Because after eruption changes in crown shape are only by wear and not through remodeling as in bones, unworn and light worn teeth directly reflect the developmental processes controlling morphogenesis and appear therefore also much more suitable for evolutionary investigations. Moreover, because very worn teeth cannot be taxonomically assigned precisely in the fossil record, they are also disqualified them from evolutionary studies as well.

The hypsodonty index H was calculated from measurements of crown height and length performed with Photoshop on virtually 3D reconstructed teeth.

## Supporting Information

Figure S1 Examples of four age/wear classes recognized in Progonomys clauzoni with their associated K value.
(0.37 MB TIF)

Figure S2 Box plot diagrams showing K and O value distribution in wear classes of Progonomys clauzoni (I, II, III, IV) and Meriones crassus.
(0.07 MB TIF)

Table S1 Comparison of average K values between Progonomys wear classes (I, II, III, IV) and Meriones.
(0.07 MB DOC)

Table S2 Comparison of average O values between Progonomys wear classes (I, II, III, IV) and Meriones.
(0.07 MB DOC)

Text S1 Relationships between tooth wear and crown topographic descriptors.
(0.07 MB DOC)
