# Supplementary figures and images for: Mosaic Convergence of Rodent Dentitions
Source: PLoS One. 2008 Oct 31;3(10):e3607. doi: 10.1371/journal.pone.0003607 (PMC2572836; doi:10.1371/journal.pone.0003607)

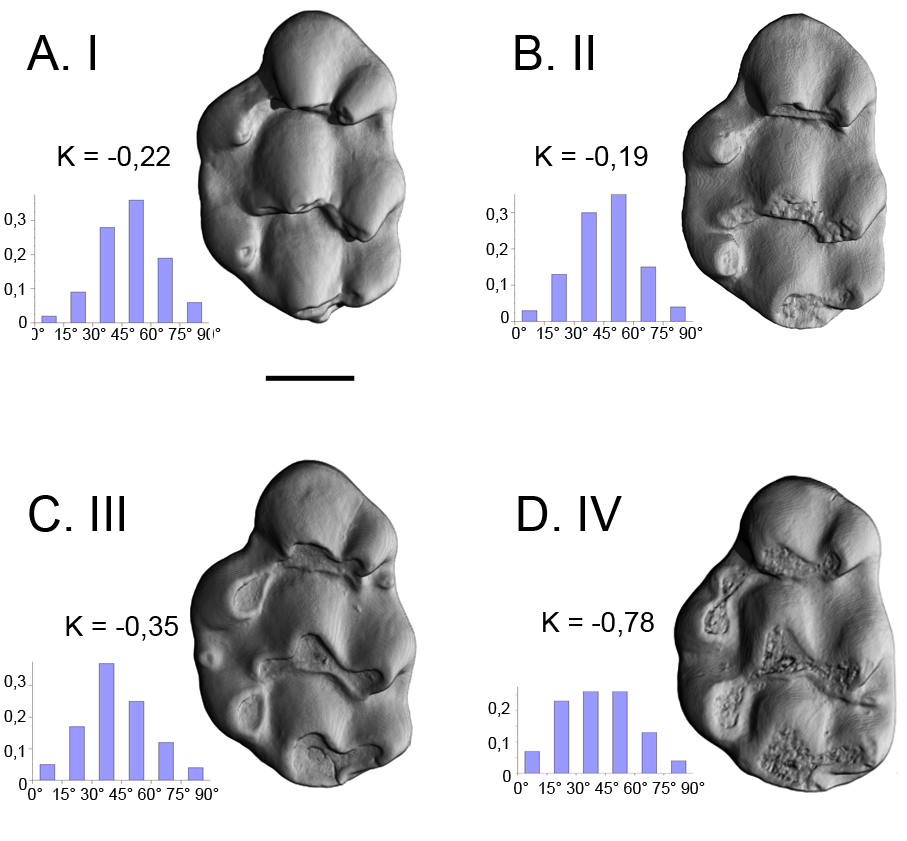

Supplement: Figure S1 — Examples of four age/wear classes recognized in Progonomys clauzoni with their associated K value. (0.37 MB TIF) [file pone.0003607.s001.tif]

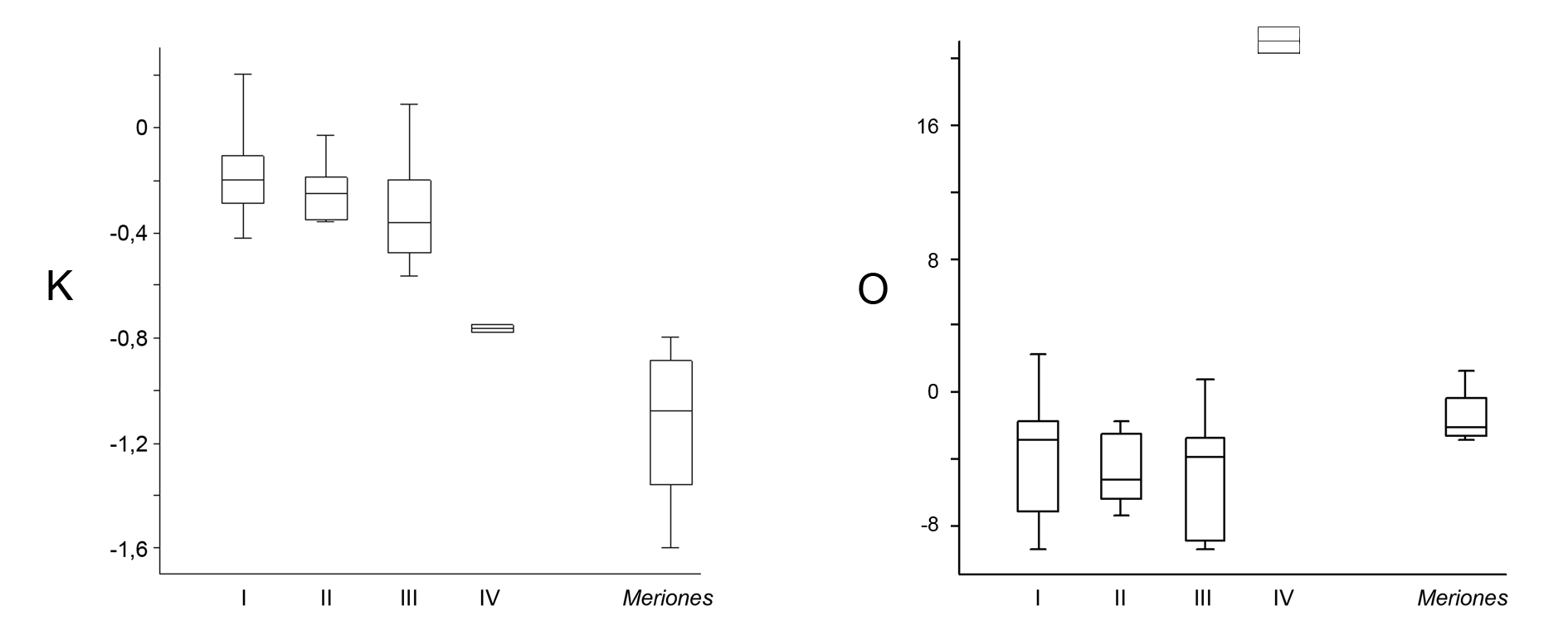

Supplement: Figure S2 — Box plot diagrams showing K and O value distribution in wear classes of Progonomys clauzoni (I, II, III, IV) and Meriones crassus. (0.07 MB TIF) [file pone.0003607.s002.tif]
