# Supplementary material for: Mosaic Convergence of Rodent Dentitions
Source: PLoS One. 2008 Oct 31;3(10):e3607. doi: 10.1371/journal.pone.0003607 (PMC2572836; doi:10.1371/journal.pone.0003607)
Supplement: Table S1 — Comparison of average K values between Progonomys wear classes (I, II, III, IV) and Meriones. (0.07 MB DOC) [file pone.0003607.s003.doc]

Table S1. Comparison of average K values between *Progonomys* wear classes (I, II, III, IV) and *Meriones*. Values associated with the Student t test of equality of means are displayed. (n = number of individuals. M = Average K value; S = standard deviation)

|  | I | II | III | IV |
| --- | --- | --- | --- | --- |
| I  (n = 10; M = -0,175; S = 0,173) |  |  |  |  |
| II  (n = 6; M = -0,238; S = 0,05) | 0,446 |  |  |  |
| III  (n = 6; M =-0,315; S = 0,096) | 0,191 | 0,493 |  |  |
| IV  (n = 2; M = -0,765; S = 0,021) | 9,3. E^-4^ | 1,16. E^-3^ | 0,042 |  |
| *Meriones*  (n = 5; M = -1,15; S = 0,332 ) | 4,02. E^-6^ | 1,47. E^-4^ | 8,91. E^-4^ | 0,186 |
