# Supplementary material for: Mosaic Convergence of Rodent Dentitions
Source: PLoS One. 2008 Oct 31;3(10):e3607. doi: 10.1371/journal.pone.0003607 (PMC2572836; doi:10.1371/journal.pone.0003607)
Supplement: Table S2 — Comparison of average O values between Progonomys wear classes (I, II, III, IV) and Meriones. (0.07 MB DOC) [file pone.0003607.s004.doc]

Table S2. Comparison of average O values between *Progonomys* wear classes (I, II, III, IV) and *Meriones*. Values associated with the Student t test of equality of means are displayed. (n = number of individuals. M = Average O value; S = standard deviation)

|  | I | II | III | IV |
| --- | --- | --- | --- | --- |
| I  (n = 10; M = -3,5; S = 3,72 |  |  |  |  |
| II  (n = 6; M = -4,83; S = 2,36) | 0,447 |  |  |  |
| III  (n = 6; M =-4,75; S = 3,93) | 0,534 | 0,965 |  |  |
| IV  (n = 2; M= 21,8; S = 2,09) | 3,77. E^-6^ | 8,14. E^-6^ | 1,18. E^-4^ |  |
| *Meriones*  (n = 4; M = -1,5; S = 1,88) | 0,333 | 0,047 | 0,167 | 1,56. E^-4^ |
