# Supplementary material for: Mosaic Convergence of Rodent Dentitions
Source: PLoS One. 2008 Oct 31;3(10):e3607. doi: 10.1371/journal.pone.0003607 (PMC2572836; doi:10.1371/journal.pone.0003607)
Supplement: Text S1 — Relationships between tooth wear and crown topographic descriptors. (0.07 MB DOC) [file pone.0003607.s005.doc]

**Text S1.** **Relationships between tooth wear and crown topographic descriptors**. If tooth wear does not alter crown topography of rodents with flattened crown because they keep a flat occlusal surface [2], it is known to dramatically alter crown topography in rodents with cuspidate crown [39,40]. Testing the influence of tooth wear on crown topographic descriptor K and O requires intra-specific analyses with individuals displaying different wear degrees. We therefore digitized in microtomography with voxel sizes of 7.46 µm 24 first upper molars of the Miocene murine *Progonomys clauzoni* and 5 first upper molars of the extant gerbil *Meriones* *crassus*. Teeth were selected in order to sample different wear degrees in both species. We computed then topographic slopes maps to measure K and O values of each tooth according to the methodology described in the Material and Methods section. Average K values were calculated for both species as the mean of the measured K values. Average K value is significantly higher in *Progonomys* (*Progonomys* average K value = -0,275; *Meriones* average K value = -1,15; Student t test: P = 1.09. E^-7^) revealing a more cuspidate topography in this species. *Progonomys clauzoni* displays cuspidate crown, while *Meriones* *crassus* displays flattened crown. Average O value is not significantly different between both taxa (*Progonomys* average O value = -3,43; *Meriones* average O value = -1,5; Student t test: P = 0,542), both displaying a propalinal chewing movement.

According to their wear the first upper molars of *Progonomys clauzoni* were classified in the age/wear classes previously established in Murinae [39,40]. Wear increases from class I to class V. Wear class I corresponds to nearly unworn teeth displaying no connexion between the dentin areas created by wear or corresponding to enamel-free areas (Fig. S1*A*). Wear class II corresponds to lightly worn teeth displaying some dentin areas that can join two-two (Fig. S1*B*). In wear class III all dentin areas join two-two and at least one longitudinal or transversal dentin area joining three cusps is present (Fig. S1*C*). In wear class IV all dentin areas are three-three connected (Fig. S1*D*). In wear class V and more, all dentin areas are connected. Distributions of K and O values of each wear class in *Progonomys* were then statistically compared (Fig. S2, Tables S1,S2). Average K and O values of classes I, II and III in *Progonomys* are not significantly different (Student t test: P > 0,05) in *Progonomys* (Tables S1,S2). However, average K value of class IV is significantly lower than average K values of classes I,II and III and is not significantly different from average K value of *Meriones* (Table S1). Average O value of class IV is also significantly higher than average O values of classes I,II,III and *Meriones* (Table S2).

These results reveals that tooth wear does significantly influence crown topography in cuspidate crowns only from class IV. From class IV threshold, crown is flattened by wear in *Progonomys*. For this study we therefore just used muroid molars displaying age/wear class I, II and III, in which wear influence on descriptors K and O is negligible.

**Figure Legends**

Fig. S1. Examples of four age/wear classes recognized in *Progonomys clauzoni* with their associated K value. The histogram of distribution of crown slopes is presented in the lower left quarter for each individual. Black scale bar: 500 µm. A: wear class I; B: wear class II; C: wear class III; D: wear class IV.

Fig. S2. Box plot diagrams showing K and O value distribution in wear classes of *Progonomys clauzoni* (I, II, III, IV) and *Meriones crassus*. A: K value distribution. B: O value distribution.
